# The Effect of Religious Attitude on Death Anxiety among Patients Undergoing Hemodialysis Treatment: A Sample from Turkey

**DOI:** 10.1007/s10943-024-02042-3

**Published:** 2024-04-15

**Authors:** Tülay Yıldırım Üşenmez, Rukiye Demir Dikmen

**Affiliations:** 1 Department of Nursing, Atatürk Health Sciences Faculty, Dicle University, Diyarbakir, Turkey; 2 Geriatric Care, Department of Medical Services and Techniques, Vocational Higher School of Health Services, Bingöl University, Bingöl, Turkey

**Keywords:** Death anxiety, Hemodialysis, Religious attitude, Renal failure

## Abstract

The aim of the current study was to evaluate the effect of religious attitude on death anxiety among patients undergoing hemodialysis treatment. This cross-sectional study included 77 patients undergoing hemodialysis treatment. The religious attitude scale (RAS) and the death anxiety scale (DAS) were used to collect data. The results revealed a moderately strong negative relationship between the mean RAS total score and the mean DAS total score (*r* = − 0.350, *p* < 0.05). Additionally, religious attitude accounted for 12% of the variance in death anxiety (*R*^2^ = 0.12). Accordingly, the levels of religious attitude exhibited by patients may negatively affect their death anxiety.

## Introduction

End-stage renal failure is a chronic disease in which the kidneys lose their function entirely. Since the kidneys cannot function, patients need routine treatment (Homaie-Rad et al., 2015). Hemodialysis (HD) is the most widely used procedure to treat end-stage renal failure in Turkey (TND Registration Reports, 2022). However, HD treatment can lead to various complications. For example, patients undergoing HD treatment may experience mental and social issues as well as physical problems (e.g., pain, suffering and various side effects) (Anjomshoa et al., 2014).

Patients undergoing HD treatment may face many stressors during the treatment process. These stressors include limitations regarding daily activities and other physical activities, dietary restrictions, changes in body image, dependence on dialysis machines, frequent hospitalization, changes in family and social relationships, and death anxiety (Anjomshoa et al., 2014; Dziubek et al., 2016; Gorji et al., 2013a, 2013b; Partridge et al., 2011; Ok & Kutlu, 2019; Sadeghian et al., 2016).

Decreased life expectancy and quality of life can lead to death anxiety among HD patients who are dependent on dialysis machines to maintain their lives (Martin & Thompson, 2000). Death anxiety can be defined as a feeling of nervousness, anxiety or fear resulting from the individual’s increased awareness of an imaginary or real threat to his or her existence (Lehto & Stein, 2009).

Even if HD treatment prolongs the lifespan of patients, living with a life-threatening disease may increase death anxiety among patients (Fathi et al., 2016). Jaberi et al. reported that the quality of life of patients undergoing HD treatment who also experienced high levels of death anxiety was low (Jaberi et al., 2022). Patients employ various strategies to cope with the difficulties caused by HD treatment. One important strategy that can help improve the health conditions of patients focuses on spirituality and religion (Hatefi et al., 2019).

A religious attitude can be defined in terms of the way in which an individual directs his or her emotions, thoughts and behavioral patterns as a result of his or her religious beliefs (Khoynezhad et al., 2012). Religious beliefs are more important during times of disease than at other times (Chakraborty et al., 2017). Patients with incurable chronic diseases may occasionally turn to religious beliefs and religious practices to relieve the symptoms they experience and to experience emotional relaxation (Luchetti et al., 2010). Valcanti et al. reported that 79.6% of chronic kidney patients undergoing HD treatment engaged in high levels of religious/spiritual coping (Valcanti et al., 2012).

Religious beliefs motivate individuals to adopt positive attitudes toward the world, face life-threatening events and increase their hope for life (Hekmati Pour & Hojjati, 2015). Since HD treatment is a long process, over time, patients’ life expectancy declines, and their motivation to adhere to therapy may decrease (Ottaviani et al., 2014). For this reason, patients undergoing HD treatment must feel good physically, mentally and socially.

Patients who have chronic diseases that require long-term treatment may need more spiritual/religious support. Nurses who care for patients undergoing HD treatment have important responsibilities in this context. Nurses must identify the religious needs of patients and plan interventions to reduce their death anxiety. Nurses should encourage patients to use their religious/spiritual resources to address the difficulties those patients experience during HD treatment. Nurses may thereby improve the quality of care they provide. The present study assumed that the religious attitude of patients undergoing HD treatment may affect their death anxiety.

The current study investigates the following questions:

- What levels of religious attitude are exhibited by patients undergoing HD treatment?
- What levels of death anxiety are exhibited by patients undergoing HD treatment?
- Is there a relationship between religious attitude and death anxiety levels among patients undergoing HD treatment?

## Methods

### Study Design and Setting

This study featured a cross-sectional design. The study was conducted in a hemodialysis unit between September and October 2022.

### Study Population and Sample

The study population consisted of 103 patients undergoing HD treatment. The aim of the current study was to focus on the entire population; thus, no sample selection method was used. Seventeen patients did not want to participate in the study. An additional nine patients were excluded from this research due to psychiatric diagnoses or renal transplants. The study ultimately included 77 patients undergoing HD treatment.

#### Inclusion Criteria

- Agreeing to participate in the study,
- Being 18 years old or older,
- Being open to communication, and
- Undergoing HD treatment

#### Exclusion Criteria

- Having been diagnosed with any psychiatric disease, dementia, neurological disease, organic mental disorder or intellectual disability
- Having received a renal transplant

### Measures

#### Information Form (IF)

This form contained eight questions (concerning the participant’s age, gender, marital status, employment status, educational status, perception of his or her own health status, time since hemodialysis treatment and cohabitants) (Ayik & Yilmaz Karabulutlu, 2020; Dewina et al., 2018).

#### Religious Attitude Scale (RAS)

The validity and reliability of the Turkish version of the scale were tested by Ok (Cronbach’s α 0.81 and 0.91) (Ok, 2011). This scale contains eight items that are scored on a five-point Likert-type scale ranging from 1 = “Strongly disagree” to “5 = Strongly agree.” Items 5 and 6 of this scale are reverse-scored. Total scores on this scale range from 8 to 40. High scores indicate high levels of religious attitude. The Cronbach’s α coefficient for this scale was 0.62 in the current study. The items included in the RAS are shown in Table 1.

**Table 1:** Items included in the RAS

Items	1 (Strongly disagree)	2	3	4	5 (Strongly agree)
1. I feel that there is no need for religion					
2. I feel that religion does more harm than good for people					
3. I feel moved when I listen to religious chants/recitations such as Ezan, prayer or Qur’anic verses					
4. I really enjoy participating in religious activities					
5. I ensure that I am living my life in line with religious values					
6. I try to put my religion into practice in my life					
7. I feel that God helps me when life is difficult					
8. I feel that God is very close to me					

#### Death Anxiety Scale (DAS)

This scale was developed by Temper (1970). The validity and reliability of the Turkish version of this scale were tested by Akça and Köse (Cronbach’s α 0.79) (Akça & Köse, 2008). The scale contains fifteen items that are scored on a two-point Likert-type scale ranging from 0 = ”No” to 1 = ”Yes.” Total scores on this scale range from 0–15. A total mean score of 7 or above indicates a high level of death anxiety. The Cronbach’s α coefficient for this scale was 0.84 in the current study.

### Statistical Analysis

The data were analyzed using IBM SPSS 25.0 software. A *p* value < 0.05 was considered to indicate significance in the current study. Internal consistency analysis of the scales was used to determine the Cronbach’s *α* coefficient. Percentile distribution, arithmetic mean, Pearson’s correlation and linear stepwise regression analyses were used to analyze the data.

### Ethical Considerations

Before starting the current study, approval was received from the Ethics Committee of X University (Approval No: 2022/16), and legal permission was acquired from the hospital at which this study was conducted. Patients were informed of the aim of the study; in addition, they were informed that their information would remain confidential and that they could cease participating in the study at any time. Additionally, the study was carried out in accordance with the principles of the Declaration of Helsinki. Written consent was obtained from the patients using a voluntary informed consent form.

## Results

A total of 49.3% of the patients were in the 62 or older age group, 88.3% were married, 53.2% were male, 40.2% were primary school graduates, 92.2% were unemployed, 44.2% perceived their health status to be “good,” 48.0% had received 0–5 years of hemodialysis treatment, and 85.7% lived with their spouse and/or children (Table 2).

**Table 2:** Distribution of the descriptive characteristics of the patients (*N* = 77)

Descriptive characteristics	*N*	%
Age groups		
29–39 years 40–50 years 51–61 years 62 years or older	9 9 21 38	11.7 11.7 27.3 49.3
Marital status		
Married	68	88.3
Single	9	11.7
Gender		
Female Male	36 41	46.8 53.2
Education level		
Illiterate	28	36.4
Primary school	31	40.2
High school or university	18	23.4
Working status		
Employed	6	7.8
Unemployed	71	92.2
Perception of own health		
Bad Moderate Good	21 22 34	27.3 28.5 44.2
Duration of hemodialysis treatment		
0–5 years 6–10 years 11–15 years 16 years or longer	37 20 12 8	48.0 26.0 15.6 10.4
Patients live with their		
Parents Spouse and/or children Other (e.g., alone or with another relative)	5 66 6	6.5 85.7 7.8
Total	77	100.0

The mean RAS total score obtained by these patients was 39.53 ± 2.09, and their mean DAS total score was 7.83 ± 2.49. Based on the means of patients’ total scores on these scales, the religious attitude and death anxiety of these patients were high (the minimum–maximum scores on these scales are 8–40 for the RAS and 0–15 for the DAS) (Table 3).

**Table 3:** Patients’ RAS and DAS scores and means

Scale	Min–max scores	Mean ± SD
RAS DAS	23–40 2–13	39.53 ± 2.09 7.83 ± 2.49

A linear stepwise regression model was used to identify the actual factors affecting death anxiety. The results of the regression analysis showed that the following factors impacted death anxiety: religious attitude, with an impact size of 0.12; religious attitude and gender, with an impact size of 0.13; religious attitude, gender and age, with an impact size of 0.17; and religious attitude, gender, age and cohabitants, with an impact size of 0.21. The total religious attitude score had a major effect on death anxiety (Table 4).

**Table 4:** Examination of the effects of religious attitude and descriptive characteristics on death anxiety using regression analysis

Dependent variable	Independent variable	Beta^b^	F	p value	R^2^	*t*	*p*
Death anxiety	1 (Constant) Religious attitude total	0.108	0.884	**0.00**	0.12	0.505 0.940	**0.00**^**a**^
	2 (Constant) Religious attitude total, gender 3 (Constant) Religious attitude total, gender, age 4 (Constant) Religious attitude total, gender, age, cohabitants	− 0.378 − 0.394 − 0.222 − 0.373 − 0.285 0.246	12.516 8.807 8.024	**0.00** **0.01** **0.00** **0.00** **0.03** **0.00** **0.00** **0.08** **0.02**	0.13 0.17 0.21	12.498 − 3.538 9.416 − 3.764 − 2.124 5.703 − 3.649 − 2.711 2.326	**0.01**^**b**^ **0.03**^**c**^ **0.02**^**d**^

Bold text *p* < 0.05

Dependent variable: death anxiety

^a^Predictors: (constant) religious attitude total

^b^Predictors: (constant) religious attitude total, gender

^c^Predictors: (constant) religious attitude total, gender, age

^d^Predictors: (constant), religious attitude total, gender, age, cohabitants

A moderately strong negative relationship was observed between the mean RAS total score and the mean DAS total score (*r* = − 0.350, *p* < 0.05). Accordingly, as patients’ levels of religious attitude increase, their levels of death anxiety decrease (Table 5).

**Table 5:** Correlation between the RAS total mean score and the DAS total mean score and the results of the correlation analysis

RAS	Correlation^⁎⁎^	
DAS	**r** *p	− 0.350 **0.000**

^*^p < 0.05 **Pearson correlation analysis

## Discussion

The results of the current study, which aimed to evaluate the effect of religious attitude on death anxiety among patients undergoing HD, are discussed by reference to the literature.

### Discussing Findings Related to Religious Attitude

The current study revealed that the levels of religious attitude exhibited by patients undergoing HD treatment are high. Religiosity and spirituality play important roles in helping individuals cope with their disease (Silva et al., 2022). Al Zaben et al., who investigated the relationship between religious practices and the health levels of patients undergoing HD treatment, reported that religious practices and internal religious beliefs were common in this context. The same study revealed that individuals who were more religious exhibited better general psychological functionality and higher levels of social support (Al Zaben et al., 2015).

Hassani et al. revealed a significant relationship between quality of life and spiritual well-being among patients undergoing HD treatment in the context of religion and existence. The quality of life of these patients increased alongside improvements in mental health and well-being (Hassani et al., 2022). Boas and Nakasu identified relationships between some dimensions of religiosity and drug compliance among patients undergoing HD treatment (Boas & Nakasu, 2021). In his study, Gencer revealed a significant relationship between religiosity and subjective well-being among patients undergoing HD treatment (Gencer, 2019).

Religious attitude can serve as a protective shield against diseases and suffering (Hojjati et al., 2015). Santos et al. reported that religious/spiritual coping methods were associated with quality of life and depression among patients undergoing HD treatment (Santos et al., 2017).

Appropriate investigations of religiosity in the context of patient care may improve adherence to HD treatment. The high level of religious attitude exhibited by patients undergoing HD treatment in our study may be due to the fact that religiosity, religious attitude and values play important roles in efforts to cope with chronic renal failure and serious diseases in our country and culture.

### Discussing Findings Related to Death Anxiety

The current study revealed that the death anxiety experienced by patients undergoing HD treatment was high. A study that investigated patients undergoing HD treatment during the COVID-19 pandemic revealed that their levels of death anxiety increased more than those of patients observed during the prepandemic period (Javed et al., 2021). In his study, Korkut revealed that the death anxiety experienced by patients undergoing HD treatment was generally moderate; however, approximately one-third of the subjects in his study exhibited high or very high levels of death anxiety (Korkut, 2022).

Dewina et al. reported that approximately half of patients undergoing HD experienced moderate death anxiety, while the other patients experienced either mild or severe death anxiety (Dewina et al., 2018). Ghiasi et al. reported that 60.4% of patients undergoing HD treatment in their study experienced high levels of death anxiety (Ghiasi et al., 2021). Khodarahimi et al., in their study of patients with chronic renal failure, showed that the mental health of these patients was positively related to death anxiety and fear of death (Khodarahimi et al., 2021). Mushtaque et al. revealed a negative effect of death anxiety on the relationship between quality of life and disease acceptance among patients undergoing HD treatment (Mushtaque et al., 2022).

The high levels of death anxiety experienced by patients undergoing HD treatment in the current study may be due to the important side effects of HD treatment as well as the many stressors that may negatively affect the physical, mental and social health of these patients.

### Discussing Findings Related to Relationship Between Religious Attitude and Death Anxiety

The current study revealed a moderately strong negative relationship between religious attitude and death anxiety among patients undergoing HD treatment. Hosseini et al., in their randomized controlled study, revealed that spirituality-focused counseling could reduce fear of death among patients undergoing HD treatment (Hosseini et al., 2022).

Based on their study, Ayik and Yilmaz Karabulutlu reported that HD patients who adopted positive religious coping styles were more likely to accept their disease (Ayik & Yilmaz Karabulutlu, 2020). Okhli et al. reported that patients undergoing HD treatment exhibited a high level of belief in religious practices and that their level of suffering decreased as these beliefs increased (Okhli et al., 2022).

Therefore, our study is important in the context of nursing. In light of the results of the current study, the hypothesis that the levels of religious attitude exhibited by patients undergoing HD treatment may affect their death anxiety was verified. For this reason, the ability of religious attitude to reduce death anxiety among patients plays an important role in routine practices that seek to help patients cope with the difficulties associated with by the disease.

No previous study has examined the effect of religious attitude on death anxiety among patients undergoing HD treatment. As such, identifying the levels of religious attitude exhibited by patients undergoing HD treatment may make a positive contribution to attempts to reduce their death anxiety.

## Limitations of this Study

The first limitation of the current study is that it was conducted in a single hospital; therefore, it focused on patients who exhibited similar cultural and social characteristics. The second limitation of this study lies in the fact that this convenience sample may not be representative of the overall population of patients undergoing HD treatment in Turkey.

## Implications for Nursing Practices

The patients included in this study exhibited high levels of religious attitude and death anxiety, and a moderately strong negative relationship was observed between religious attitude and death anxiety in this context. At present, HD treatment is the most widely used method to treat end-stage renal failure in Turkey. Scholarly interest in the use of religious attitude to address critical diseases associated with difficult treatment processes such as HD is increasing.

Religious attitude plays a significant role in the ability of HD patients to adapt the treatment process. In addition, religious attitude may be beneficial with regard to coping with the negative effects of the death anxiety experienced by patients undergoing HD treatment. Thus, nurses should integrate spiritual/religious resources into patients’ care. Interventions that include spiritual/religious therapies, group counseling, cognitive therapy, education regarding hemodialysis procedures and health issues related to chronic renal failure can be used to reduce death anxiety.
